# Supplementary material for: Interconnectedness enhances network resilience of multimodal public transportation systems for Safe-to-Fail urban mobility
Source: Nat Commun. 2023 Jul 18;14:4291. doi: 10.1038/s41467-023-39999-w (PMC10354098; doi:10.1038/s41467-023-39999-w)
Supplement: Supplementary file 1 — Supplementary Information [file 41467_2023_39999_MOESM1_ESM.pdf]

Supplementary information

Interconnectedness enhances network resilience of multimodal public transportation systems for *Safe-to-Fail* urban mobility

Zizhen Xu, Shauhrat S. Chopra*
School of Energy and Environment, City University of Hong Kong, Tat Chee Avenue, Hong Kong SAR

Email: S.S.C. <sschopra@cityu.edu.hk>, X.Z. <zizhenxu2@cityu.edu.hk>

Supplementary Figures

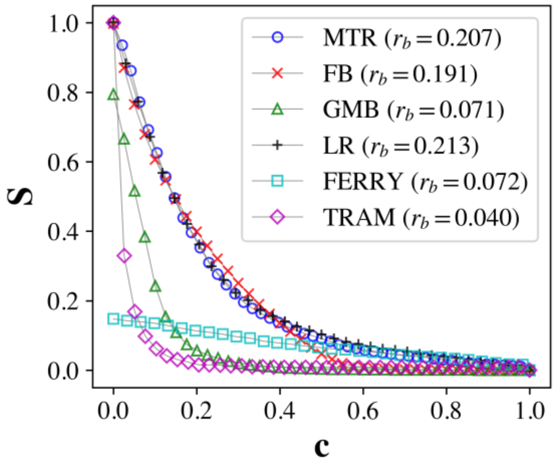

(a) Random disruptions.

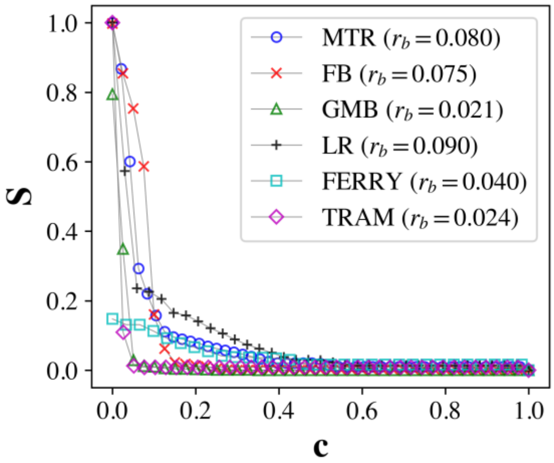

(b) Node degree-targeted attacks.

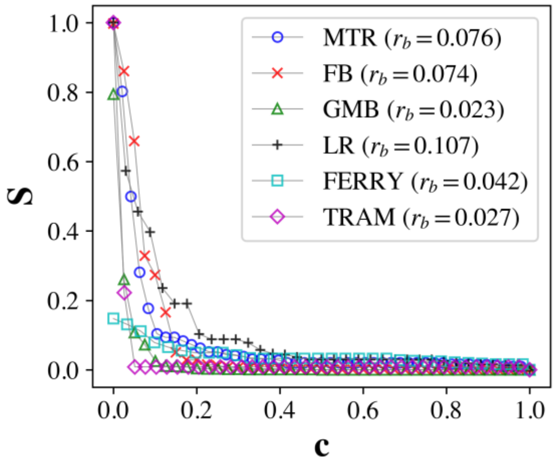

(c) Node betweenness-targeted attacks.

Supplementary Fig. 1 Robustness of sub-networks. The degradation curves are shown for the scenarios of **(a)** random disruptions, **(b)** node-degree targeted attacks, and **(c)** node betweenness-targeted attacks. MTR, LR, and FB networks show better robustness in all three scenarios, with high $\langle k_{out} \rangle$ contributing to network redundancy. In the random failure scenario, MTR and LR have similar degradation trends, while LR performs better than MTR in targeted attacks. FERRY, TRAM, and GMB networks exhibit poor robustness due to low $\langle k_{out} \rangle$. Surprisingly, GMB performs worse than TRAM in targeted attacks though the former has a higher $\langle k_{out} \rangle$., likely due to its high heterogeneity, which makes the structure susceptible to fracturing by attacks on critical nodes.

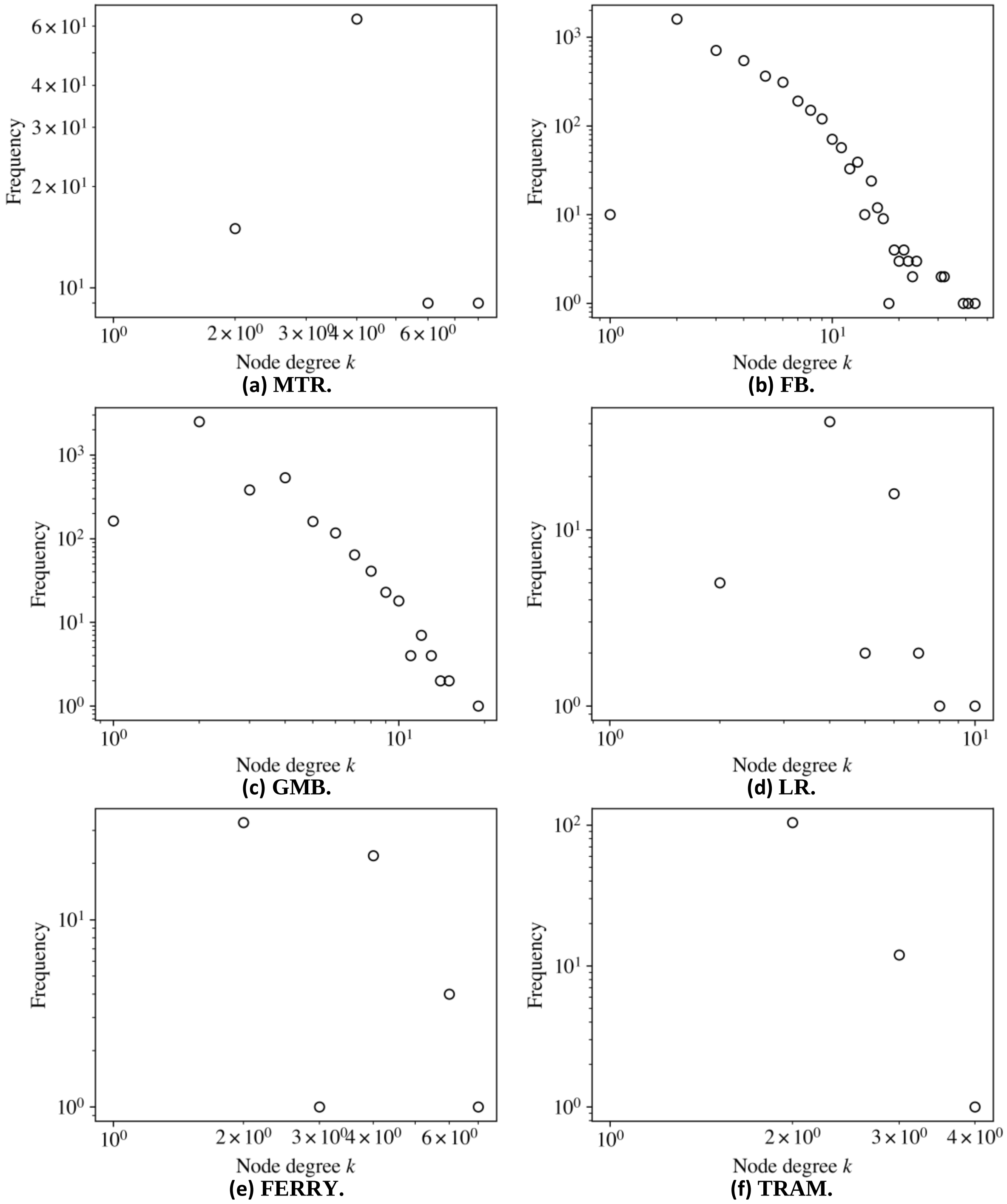

Supplementary Fig. 2 Degree distribution of each subnetwork. The summed number of in- and out-degree is adopted. Examining the degree distribution, the FB and GMB bus systems show identifiable heavy tails, suggesting they are more robust to random failures but more vulnerable to targeted attacks than random networks. Other subsystems have mostly unidentifiable trends.

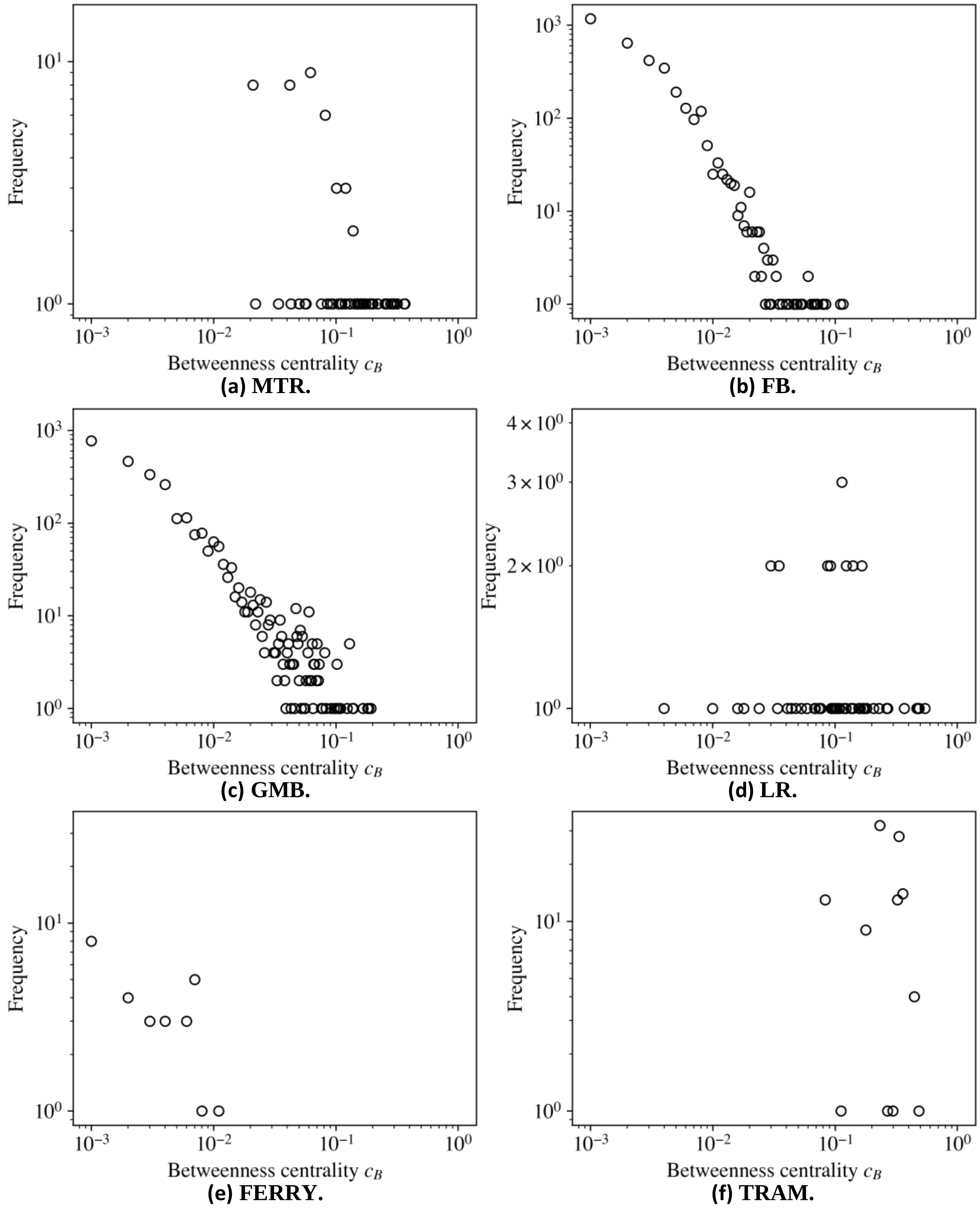

Supplementary Fig. 3 Distribution of node betweenness centrality. Note that betweenness centrality values are normalized by 1/((n-1) (n-2)) for directed graphs where n is the number of nodes in the graph. Regarding node betweenness, the MTR, LR, and TRAM systems have a concentrated distribution around medium betweenness value, meaning most nodes have betweenness level close to each other. Such similarity may result from a similar design purpose. In contrast, the heavy-tail distribution in the GMB and FB networks leads to vulnerabilities from attacks on high-betweenness stations where passenger flows concentrate.
